# Editorial: Health data science and AI in neuroscience & psychology

**DOI:** 10.3389/fncom.2026.2002192

**Published:** 2026-10-05

**Authors:** Lei Lu, Zhiqiang Huo, Jin Li, Erying Zhao

**Affiliations:** 1 School of Life Course & Population Health Sciences, King's College London, London, United Kingdom; 2 Wolfson Institute of Population Health, Queen Mary University of London, London, United Kingdom; 3 Jiangsu Key Laboratory of Intelligent Medical Image Computing, School of Artificial Intelligence, Nanjing University of Information Science and Technology, Nanjing, China; 4 Psychology and Health Management Center, Harbin Medical University, Harbin, China

**Keywords:** AI, depression, health data science, mental health, neuroscience, psychology

Health data science and artificial intelligence (AI) are reshaping neuroscience and psychology, by enabling the integration of functional, biological, clinical, and behavioral data, and by advancing the analytics applied to them, yielding insights that are difficult to obtain through conventional approaches alone (Gong et al., 2025, Salemi et al., 2025). However, such data from these domains are often noisy, heterogeneous, and unevenly labeled, complicating model development, result interpretation, and clinical translation (Gell et al., 2024). This Research Topic, Health Data Science and AI in Neuroscience & Psychology, brings together studies from diverse methodological and disciplinary perspectives that address these data challenges. Across contributed publications, this Research Topic covers deep learning, mental-health understanding, clinical decision support, rehabilitation technology, and comprehensive review of AI models in neurodegenerative diseases. As discussed below, these contributions showcase recent progress of health data science and AI in bridging complex multimodal data and meaningful insights for advancing neuroscience and psychology. They also underscore the need for robust, interpretable, and clinically relevant approaches that are generalizable across populations and real-world settings.

Gao et al. introduced a deep learning framework for health risk assessment in adolescents by developing a combination of a two-dimensional convolutional autoencoder (2DCNN-AE) with multi-sequence learning and multi-scale asynchronous correlation information extraction techniques. Applied to modeling fMRI data from the Adolescent Risk Behavior (AHRB) dataset, the framework was designed to capture both spatial and time-shifted relationships among brain regions considering limited labeled data. The study demonstrated the promising performance of a deep learning-based approach for understanding and predicting health-related risks in adolescents. The work illustrated the potential of the developed deep learning method in enhancing the precision of health risk assessment and supporting early identification of individuals who may benefit from timely intervention. However, more robust external validation and assessment of generalizability across diverse populations and settings are essential to establish the clinical utility and readiness for real-world implementation.

Chalmers et al. developed a study of using deep reinforcement learning to simulate the effect of dendritic spine loss as observed in major depressive disorder (MDD). The simulated spine loss produced a variety of behaviors in the artificially intelligent agent, such as anhedonia, increased temporal discounting, avoidance, and an altered exploration/exploitation balance. Furthermore, simulating alternative and longstanding reward-processing-centric conceptions of MDD (dysfunction of the dopamine system, altered reward discounting, context-dependent learning rates, increased exploration) did not produce the same range of MDD-like behaviors. This study highlighted the potential of AI as a powerful tool for investigating underlying mechanisms beyond diagnosis and outcome prediction, providing a computational approach to probe the pathophysiology of MDD and inform hypotheses for potential therapeutic strategies.

Haykal and Kroumpouzos contributed a perspective in examining opportunities, limitations, and ethical considerations of leveraging AI in detecting body dysmorphic disorder (BDD), a frequently overlooked yet clinically significant condition. BDD characterized by a persistent and intrusive preoccupation with perceived flaws in appearance, typically minor or imperceptible to others. Individuals with BDD often seek repeated cosmetic procedures in hopes of achieving relief, but they typically find little psychological benefit from such interventions. In this perspective, the authors explored the potential role of using AI approaches in improving the management of BDD, such as early diagnosis, prompt reflection, highlight concerning patterns, and enhancing clinical sensitivity, considering transparency, empathy, and restraint.

Human-machine interaction and computational neuroscience also provide promising opportunities to advance rehabilitation, particularly for older adults (Lu et al., 2021). In the work from Xing et al., a deep learning model was developed to process point-cloud data acquired from laser sensors for non-contact analysis of hand gestures and surface features, with the aim of supporting hand function rehabilitation with human-machine interaction. By analyzing hand surface point clouds, local feature extraction, and abstraction and enhancement of dimensional information, the proposed approach effectively captured relevant spatial information and demonstrated promising performance in gesture and hand-surface feature recognition. These findings highlight the potential of combining AI with non-contact sensing technologies to support more natural, accessible, and data-driven approaches to rehabilitation and human-machine interaction.

Finally, Liu et al. reviewed the application of AI-based radiogenomics in managing neurodegenerative diseases, such as Multiple Sclerosis (MS), Dementia, Parkinson's Disease (PD), Amyotrophic Lateral Sclerosis (ALS), and Creutzfeldt-Jakob Disease. The study summarized representative AI models in neurodegenerative diseases, a range of publicly available data resources, supporting radiomics, genomics, and radiogenomics studies. It also highlighted key directions for future research in computational modeling for neurodegenerative diseases, including strategies to mitigate overfitting in high-dimensional genomic data, robust validation approaches that account for modality-specific variability and incomplete data, and efforts to bridge the gap between AI-enhanced models and their translation into real-world clinical practice.

Looking forward, health data science and AI are rapidly evolving fields, with emerging methodologies providing opportunities beyond those demonstrated in this Research Topic. For example, large language models may enable more sophisticated analysis and interpretation of complex information, supporting personalized risk assessment and tailored intervention (Li et al., 2026), while multimodal learning could integrate complementary data sources to generate more comprehensive representations of individual health (Liu et al., 2025). Integrating subjective (e.g., questionnaires) and objective measurements (e.g., wearable sensing data) may further enhance the understanding of individual health (Lim et al., 2024). Further advances in model optimization, robustness, interpretability, and clinical validation will be essential for translating these innovations into real-world applications (Shen et al., 2024). Ultimately, interdisciplinary synergy across neuroscience, psychology, clinical medicine, and computational sciences will be critical to developing AI systems that are technically capable, clinically meaningful, and generalizable across diverse populations and settings. We hope this Research Topic will stimulate further interdisciplinary research and responsible translation of health data science and AI in neuroscience and psychology.
